# Stability of simultaneously placed dental implants with autologous bone grafts harvested from the iliac crest or intraoral jaw bone

**DOI:** 10.1186/s12903-015-0156-x

**Published:** 2015-12-30

**Authors:** Young-Hoon Kang, Hyun-Min Kim, June-Ho Byun, Uk-Kyu Kim, Iel-Yong Sung, Yeong-Cheol Cho, Bong-Wook Park

**Affiliations:** Department of Oral and Maxillofacial Surgery, Gyeongsang National University School of Medicine, Institute of Health Science, Jinju, 660-702 Republic of Korea; Department of Oral and Maxillofacial Surgery, School of Dentistry, Pusan National University, Busan, Republic of Korea; Department of Oral and Maxillofacial Surgery, College of Medicine, Ulsan University, Ulsan, Republic of Korea

**Keywords:** Simultaneous dental implantation, Severely atrophic alveolar ridge, Autologous bone graft, Iliac bone, Intraoral jaw bone

## Abstract

**Background:**

Jaw bone and iliac bone are the most frequently used autologous bone sources for dental implant placement in patients with atrophic alveolar ridges. However, the comparative long-term stability of these two autologous bone grafts have not yet been investigated. The aim of this study was to compare the stability of simultaneously placed dental implants with autologous bone grafts harvested from either the iliac crest or the intraoral jaw bone for severely atrophic alveolar ridges.

**Methods:**

In total, 36 patients (21 men and 15 women) were selected and a retrospective medical record review was performed. We compared the residual increased bone height of the grafted bone, peri-implantitis incidence, radiological density in newly generated bones (HU values), and implant stability using resonance frequency analysis (ISQ values) between the two autologous bone graft groups.

**Results:**

Both autologous bone graft groups (iliac bone and jaw bone) showed favorable clinical results, with similar long-term implant stability and overall implant survival rates. However, the grafted iliac bone exhibited more prompt vertical loss than the jaw bone, in particular, the largest vertical bone reduction was observed within 6 months after the bone graft. In contrast, the jaw bone graft group exhibited a slower vertical bone resorption rate and a lower incidence of peri-implantitis during long-term follow-up than the iliac bone graft group.

**Conclusions:**

These findings demonstrate that simultaneous dental implantation with the autologous intraoral jaw bone graft method may be reliable for the reconstruction of edentulous atrophic alveolar ridges.

## Background

Over the past several decades, numerous new dental implant materials and techniques have been introduced in an attempt to increase the survival rates of placed implants. However, the most serious obstacle in dental implantation is atrophic alveolar ridges. When patients have atrophic alveolar ridges, their implant success rates decrease significantly compared with patients that have thick alveolar ridges [1]. Various bone graft techniques have been developed to enhance alveolar bone volume and height for successful implantation in atrophic ridges. There are various factors to be considered in the selection of graft material and in the determination of optimal implant placement time. These include autologous bone versus allogenic or synthetic bone, block bone versus particulate bone, donor site selection for autologous bone harvesting, and immediate versus delayed implant placement.

There is still controversy relating to whether implant placement should be performed immediately or if it should be delayed for a period of time after bone graft. In patients with less than 4 mm residual bone height in the maxillary posterior ridge, delayed implant placement at 6 to 18 months after subantral bone grafting is highly recommended [2, 3]. However, other researchers have reported similar implant success rates between delayed and immediate implantation after bone graft in the maxillary posterior ridge in patients’ exhibiting a residual bone height of less than 4 mm [4]. Similarly, many other studies have also shown high survival rates for immediately placed implants with various bone graft techniques in severely atrophic alveolar ridges [5–8].

Autologous bone for alveolar ridge enhancement can be harvested from various sites such as the ilium, the tibia, the fibula, the calvaria, and the intraoral jaw bone. The intraoral jaw bone is defined as the bone harvested from the maxilla and the mandible that usually includes the chin (mandibular symphysis and parasymphysis), the mandibular ramus (external oblique ridge), and the maxillary tuberosity. The jaw bone can usually be easily harvested from the oral cavity in the area surrounding the surgical field of implant placement, without the need of secondary surgery for bone harvesting. The iliac bone is also widely utilized as an autologous bone source for the reconstruction and the augmentation of jawbones. Jaw bone and iliac bone are the most frequently used autologous bone sources for dental implant placement in patients with atrophic alveolar ridges. However, the comparative long-term stability of these two autologous bone grafts, including the prognosis of dental implants placed in the grafted bones, have not yet been investigated.

The aim of this study was to compare the stability of simultaneously placed dental implants with autologous bone grafts harvested from either the iliac crest or the intraoral jaw bone for severely atrophic alveolar ridges. We compared the residual increased bone height of the grafted bone, incidence of peri-implantitis, radiological density in newly generated bones, and implant stability using resonance frequency analysis between the two autologous bone graft groups.

## Methods

### Patient selection

A total of 36 patients (21 men and 15 women) were selected for this study and a retrospective review of their medical records was performed. Informed consent for the use of preoperative and postoperative data was obtained from all patients, and this study was approved by the Ethics Committee for Clinical Research at Gyeongsang National University Hospital. The inclusion criteria were patients who agreed to participate in the study and who had completed at least 3 years of follow-up after undergoing simultaneous dental implantation and autologous bone grafting (with grafts harvested from either the iliac crest or the intraoral jaw bone) for the reconstruction of partially or fully edentulous upper and/or lower alveolar ridges. The donor site was selected according to the surgeon’s consideration of required bone quantity on a per case basis. We excluded patients who (1) had undergone surgery for implant-supported overdenture, (2) received implants after tumor resection, (3) had been treated with bisphosphonates, and f had been followed up for less than 3 years.

### Surgical procedures

All patients underwent simultaneous dental implant placement with autologous bone grafts under general anesthesia. They were divided into two groups based on the bone graft donor site: the iliac bone (Group 1) and the intraoral jaw bone (Group 2). The iliac bone was harvested from the iliac crest through a trap door opening, as previously described [9]. The intraoral jaw bone was harvested from the chin, the mandibular ramus (external oblique ridge), and/or the maxillary tuberosity.

**Fig. 1:**
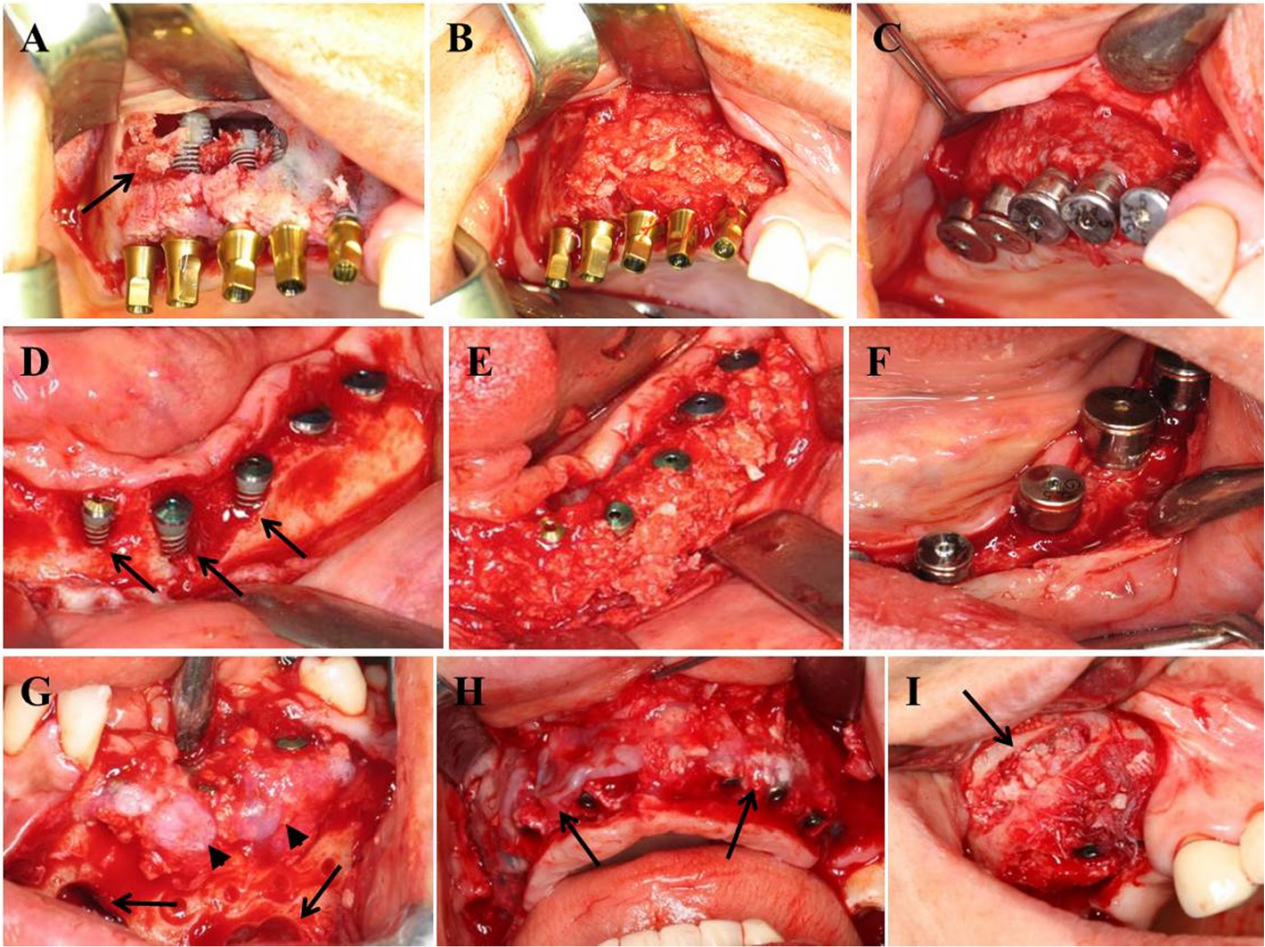
Images show the simultaneous dental implantation with autologous iliac bone and intraoral jaw bone grafting procedure. **a–c** Dental implant fixtures are placed with inlay type iliac bone grafts in the maxillary sinus. **a** The iliac block bone (arrow) is grafted into the sinus floor and fixed with implant fixtures. **b** The dead space in the sinus floor is filled with mixed bone of autologous particulate iliac bone and demineralized bone matrix (DBM). **c** The initial bone healing is completed with homogeneous new bone formation around fixtures 6 months postoperatively. **d–f** Dental implantation with onlay type bone grafts for coverage of the exposed fixtures using autologous iliac particulate bones. **d** Partial exposure of implant fixtures is viable after implant placement on the irregular mandibular ridge (arrows). **e** The exposed fixtures are covered with a mixture of particulate iliac bone and DBM. **f** The grafted bone heals with new bone formation 5 months after bone graft. **g–i** Photographs show autologous jaw bone grafts, both of onlay- and inlay-type, for simultaneous implantation. **g** Autologous chin bone is harvested (arrows indicate chin bone harvested sites) and crushed into particulate, then onlay-type grafted for the exposed fixtures in the mandibular ridge (arrowheads indicate fibrin glue injection on particulate jaw bone graft site). **h** In the maxillary ridge, the exposed fixtures are covered with a mixed bone of particulate jaw bone and DBM (arrows). **i** Maxillary sinus windows are opened and sinus membrane elevated (arrow), the mixed bone of jaw bone and DBM is subantral inlay-type grafted after placement of implant fixtures

The edentulous alveolar ridges were exposed with alveolar crest incisions. In the posterior maxilla, the lateral window was opened, and the sinus mucosa was elevated, as previously described [10, 11]. The submerged types of dental implants (BioHorizon™, BioHorizon Implant System, AL, USA; Osstem™, Osstem Implant Co., Seoul, Korea) were placed according to previously calculated positions and depths using surgical stents. The harvested iliac block bone was contoured for transplantation in the sinus floor (subantral inlay block bone graft) to increase initial stabilization of placed implant fixtures (Fig. 1a). Other harvested autologous bone from the ilium or the intraoral jaw bone was reduced to particulate chips and mixed with a demineralized bone matrix (DBM; Bongener™, CGBio Co., Seongnam, Korea), with a volumetric ratio that was two-thirds autologous bone and one-third DBM (v/v ratio: 2:1) for each group, for onlay- and/or inlay-types of bone graft. A mixture of autologous bone and DBM was grafted onto the ridge to cover the implanted fixtures (onlay graft) and transplanted into the sinus floor to fill the cavity between the sinus floor and the membrane (inlay graft) (Fig. 1). Fibrin glue (Greenplast™, Green cross, Yongin, Korea) was injected onto the grafted bones, and covered with an absorbable membrane (CollaGuide™, Bioland Co., Chengwon, Korea). The surgical sites were closed with 3/0 silk. At 5 to 6 months post-simultaneous implant placement with autologous bone graft, the surgical fields were reopened and the healing abutments were connected onto the placed fixtures (Fig. 1c & f). Patients received fixed prostheses with metal or gold ceramic crowns and bridges.

### Clinical and radiological analysis of dental implant stability

**Fig. 2:**
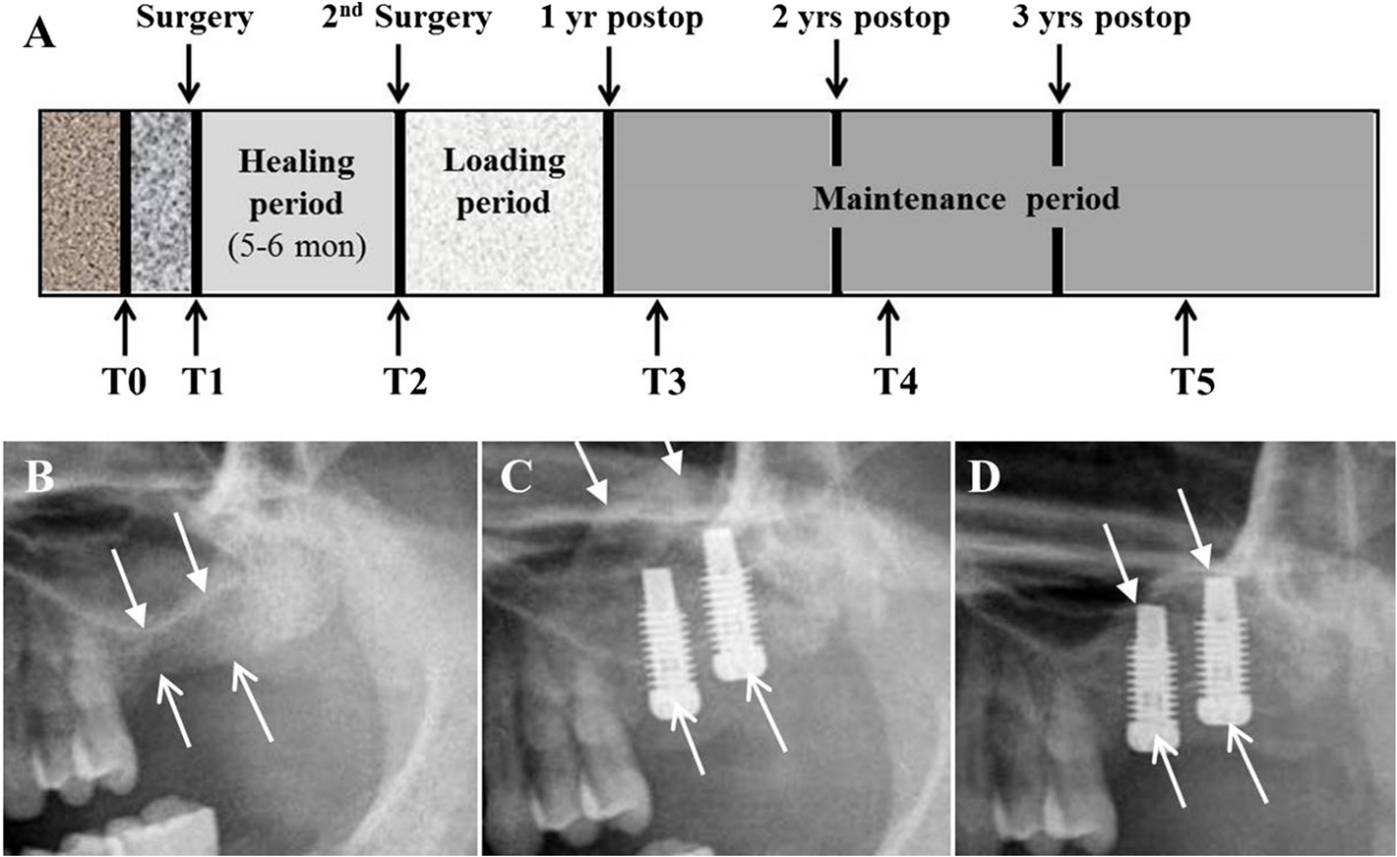
**a** Schematic diagram shows the specific time points for the measurements and analyses in the present study. **b–d** Images show the method for residual bone height measurement in panoramic views. **b** Patient shows a thin residual bone height at the preoperative panoramic view (arrows) (T0). **c** Immediately after simultaneous implant placement with iliac bone grafting (T1), the alveolar bone height increases due to the graft material (arrows). **d** Five months after surgery (T2), the subantral inlay-type grafted bone volume is shrunk and elevated sinus floor is inferiorly moved because of bone healing and consolidation

**Fig. 3:**
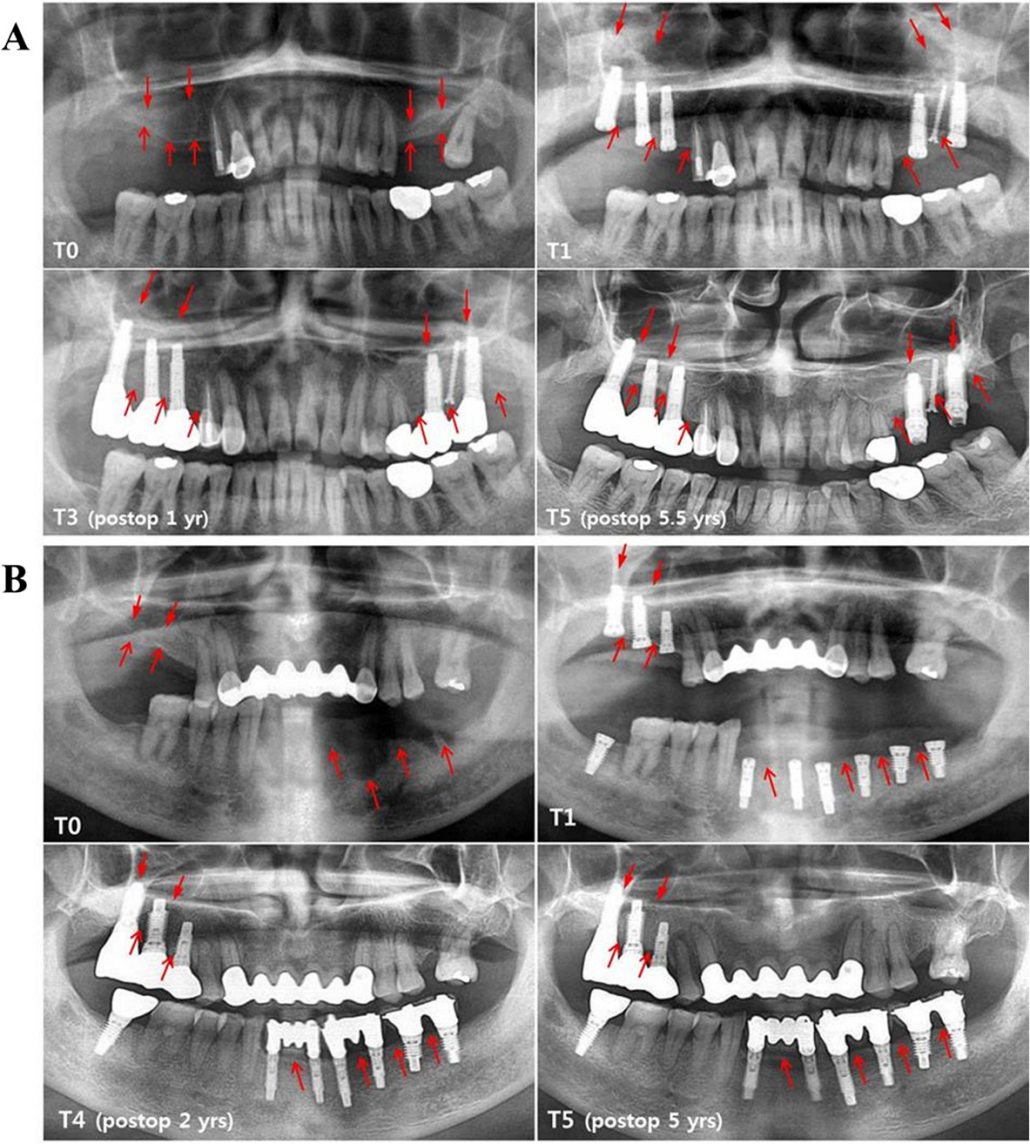
Long-term panoramic evaluation of simultaneous dental implantation cases with autologous iliac bone (**a**) and intraoral jaw bone grafts (**b**). **a** Dental implants are placed in both maxillary posterior ridges with subantral inlay-type iliac block and particulate bone graft. In panoramic analysis, the augmented alveolar bone heights in both maxillary posterior ridges (arrows in T3) are remarkably vertically reduced at the 5.5-year follow-up (arrows in T5). In particular, the radiograph of T5 (5.5 years postoperatively) shows coincidental bone resorption in the marginal alveolar bone (open arrows) and sinus floor (closed arrows) compared with radiographs at T1 or T3, indicating that the long-term grafted bone resorption could be related to the shrinkage volume of grafted iliac bone as well as peri-implantitis. **b** A case of intraoral jaw bone graft and simultaneous implantation. Implant fixtures are simultaneously placed and jaw bone is grafted onto the sinus floor (subantral inlay-type) and on the exposed fixtures in lower alveolar ridges (onlay-type). The grafted jaw bone is well maintained and shows a lesser vertical bone reductive pattern than the iliac bone graft in the marginal alveolar bone (open arrows) and sinus floor (closed arrows)

**Fig. 4:**
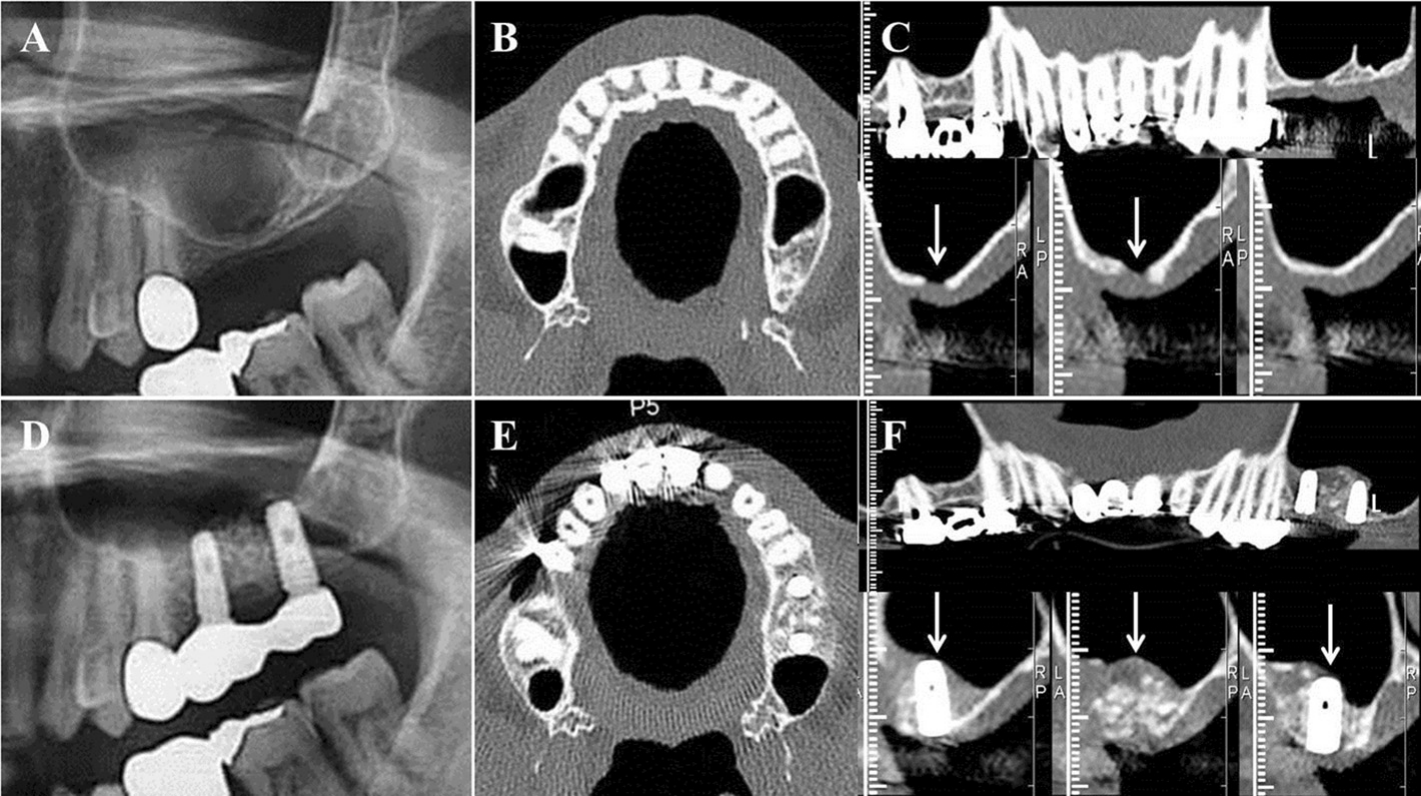
Panoramic and computerized tomographic (CT) evaluation of the subantral inlay jaw bone graft site. **a–c** Preoperative panoramic and CT views (axial, coronal, and sagittal) show a thin alveolar bone height in left maxillary posterior ridge, even perforation of alveolar bone is observable (arrows). **d–f** Radiographs at 6 months after surgery (T3) showed the stabilization of implant fixtures with newly generated bone in the maxillary sinus floor (arrows)

We evaluated preoperative and the sequential postoperative radiological views to calculate the residual vertical bone height of each group. Routine panoramic views were taken immediately before surgery (T0), immediately after implant placement and bone grafting (T1), immediately before reopening the placed fixtures (second implant surgery) at 5 to 6 months after bone graft (T2), and then annually at the follow-up periods (T3 to T5): T3, between 1 and 2 years after surgery; T4, between 2 and 3 years after surgery; and T5, more than 3 years after surgery (Fig. 2a). In serial panoramic views of the inlay- and onlay-type bone graft sites, the vertical alveolar bone height was measured and calculated, and the residual increased bone height was compared with the preoperative vertical alveolar bone height (T0) (Fig. 3). The ratio of residual grafted bone height was calculated at T5 by comparing the initial increased bone height at T1: [(remaining grafted bone height at T5)∕(initial increased bone height at T1) × 100] (Table 2). Dental computed tomography (CT) scans (Philips Medical System, Ohio, USA) were taken in 25 consenting patients (15 in Group 1 and 10 in Group 2) 1 year postoperatively (T3) (Fig. 4). From the CT scans, radiological intensities were analyzed by measurements of HU values in the newly generated bones using image analyzing software (Syngo CT 2004A, Siemens, Munich, Germany) and compared between the two groups.

The implant stability quotients (ISQ) were measured by Osstell™ Mentor (Osstell, Gothenburg, Sweden) during the second implant surgery procedure at 5 to 6 months after fixture placement (T2) (Fig. 6). The ISQ was measured at least three times for each fixture, and was represented as the mean ± standard deviation (SD) of both the subantral inlay-type and the onlay-type bone graft groups. For all fixtures, the incidence of peri-implantitis was analyzed by probing pocket depth (PPD) and bleeding on probing (BOP) during the annual follow-up periods (T3 ~ T5). The data were digitalized and statistically evaluated between the two groups.

### Statistical analysis

All data for residual increased bone height, ISQ value, HU value, and peri-implantitis indexes were represented by mean ± SD at each time point of each group. The statistical differences between Groups 1 and 2 were determined using one-way analysis of variance, followed by the Tukey test for multiple comparisons, or the unpaired *t*-test for single comparisons of experimental data between the two groups, using GraphPad Prism analysis software (GraphPad Software, San Diego, CA, USA). All statistical results were considered significant at *p* < 0.05, and these differences were denoted by an asterisk or by different letters.

## Results

### Patient information

A total of 368 implant fixtures in 36 patients were simultaneously placed with autologous bone grafts: 193 fixtures were implanted with iliac bone graft (Group 1) in 20 patients (11 men and nine women) while 175 fixtures were placed with intraoral jaw bone graft (Group 2) in 16 patients (ten men and six women). Patient age was between 40 and 72 years, with a mean age of 56.2 ± 9.5 years (Group 1: 59 ± 8 years; Group 2: 53 ± 10 years). A total of 225 fixtures were placed in the maxilla and 143 implants were placed in the mandible. Among the maxillary implants, 120 fixtures (Group 1: 77 fixtures in 18 patients; Group 2: 43 fixtures in 14 patients) were placed in the maxillary sinus with subantral inlay bone graft using the lateral window technique. The other 248 fixtures (Group 1: 116 fixtures in 18 patients; Group 2: 132 fixtures in 15 patients) were placed with onlay-type bone grafts in the maxillary and the mandibular residual ridges. No implant showed early osteointegration failure at the T2 stage, even though some fixtures showed partial exposure of their labial threads due to volume shrinkage of the graft materials. However, in the subantral placed implants, four and three fixtures were lost in Groups 1 and 2, exhibiting 94.8 and 92.7 % survival rates, respectively. Similarly, in the onlay-type bone graft sites, Groups 1 and 2 lost two fixtures each, showing 98.3 and 98.5 % survival rates, respectively. Further information on the placed fixtures with their sites and success rates is shown in Tables 1 and 2.

**Table 1:** Number of implants placed simultaneously with autologous bone grafting, classified by fixture type and placement site

		Anterior		Premolar		Posterior		Total
		BioH	Osst	BioH	Osst	BioH	Osst	
Group 1 (Ilium + DBM)	Mx	14	11	24	9	45	18	121
	Mn	8	7	9	5	27	16	72
Group 2 (MMB + DBM)	Mx	8	27	11	11	24	23	104
	Mn	4	13	8	13	13	20	71
Total		34	58	52	38	109	77	368

Abbreviation: *Mx* maxillary arch, *Mn* mandibular arch, *BioH* BioHorizon fixtures (BioHorizon™, BioHorizon Implant System, AL, USA), *Osst* Osstem fixtures (Osstem™, Osstem Implant Co., Seoul, Korea), *MMB* maxillomandibular bone, *DBM* demineralized bone matrix (Bongener™, CGBio Co., Seongnam, Korea)

**Table 2:** Implant survival rate at all follow-up period and the ratio of residual grafted bone height at T5

		No. of patients/No. of fixtures	Implant Failure			Overall Implant Survival Rate*	Ratio of Residual Grafted Bone Height at T5**
			Early (~T2)	Middle (T2 ~ T4)	Late (T5~)		
Subantral Inlay Graft	Gr1	18/77	0	3	1	94.8 %	51.9 %^a^
	Gr2	14/43	0	2	1	93.0 %	76.0 %^b^
	Total	32/120	0	5	2	94.2 %	62.8 %
Onlay Graft	Gr1	18/116	0	1	1	98.3 %	53.1 %^a^
	Gr2	15/132	0	2	0	98.5 %	75.6 %^b^
	Total	33/248	0	3	1	98.4 %	63.6 %

*There is no statistically significant difference in implant failure rate and overall implant survival rate between the two groups (*p* > 0.05)

**The ratio of residual grafted bone height was calculated at T5 by comparing the initial increased bone height at T1: [(remaining grafted bone height at T5)∕(initial increased bone height at T1) × 100]

^**a,b**^: different letters indicate the statistical difference between Group 1 and Group 2 (*p* < 0.05)

### Radiological analysis

**Fig. 5:**
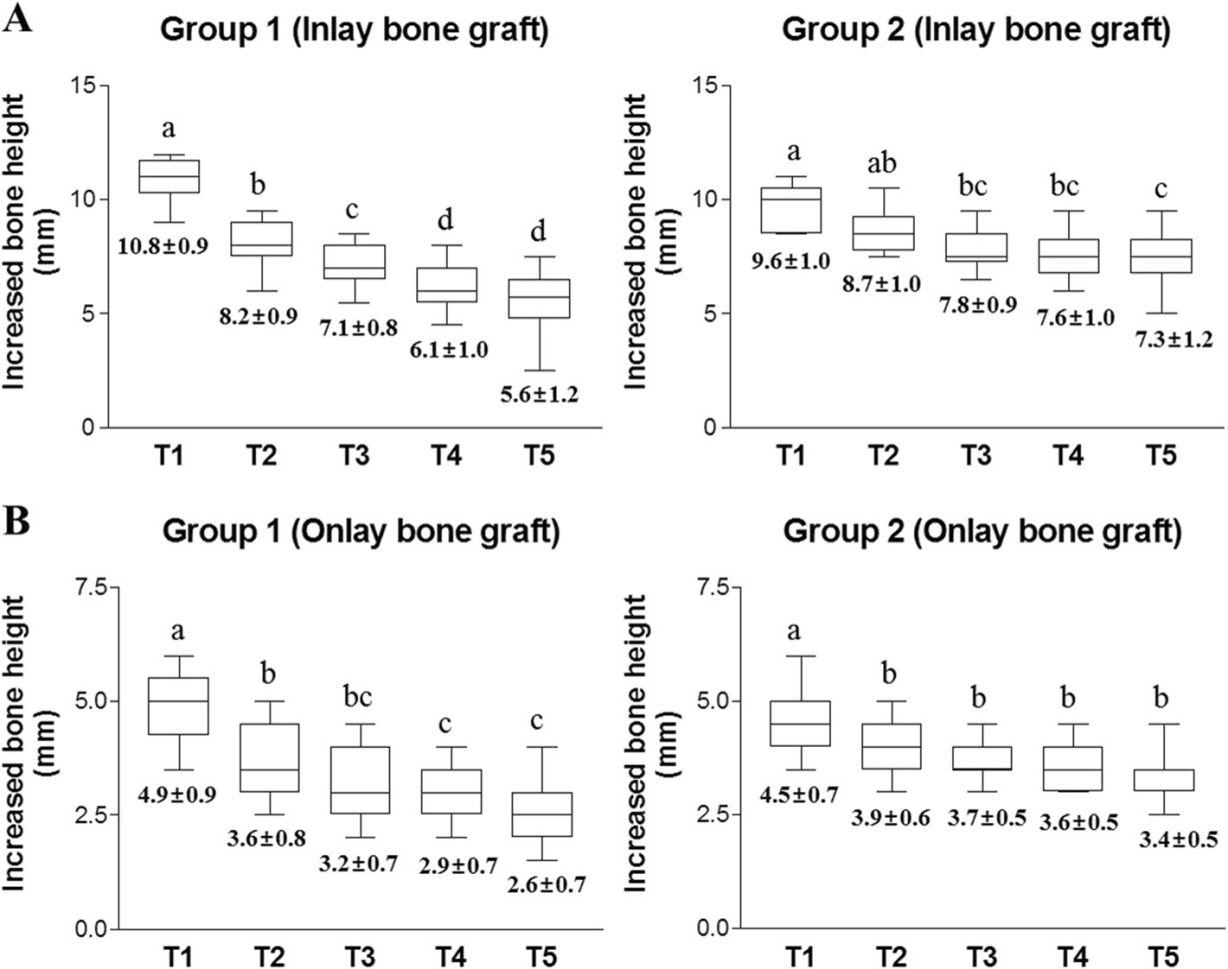
Box plots showing changes in mean vertical increased bone height in the panoramic views at each time point. **a** In subantral inlay type bone graft sites, Group 1 shows a greater reduction of residual bone height than Group 2. In Group 1, the mean increased vertical bone height is 10.8 ± 0.9 mm immediately after the operation (T1), but it promptly decreases to 8.2 ± 0.9 mm 6 months postoperatively (T2), and continuously reduces to 5.6 ± 1.2 mm by T5. However, in Group 2, there is no statistical difference in the mean increased bone height between T1 (9.6 ± 1.0 mm) and T2 (8.7 ± 1.0 mm), and this gradually decreases to T5 (7.3 ± 1.2 mm). **b** Onlay type bone graft sites show similar mean residual bone height changes to the inlay type bone graft sites. Group 1 exhibit more rapid vertical bone loss than Group 2. In both groups, the largest bone loss occurs between T1 and T2. Data represent mean ± standard deviation of increased vertical bone height at each time point, and different letters denote statistically significant differences (*p* < 0.05)

**Fig. 6:**
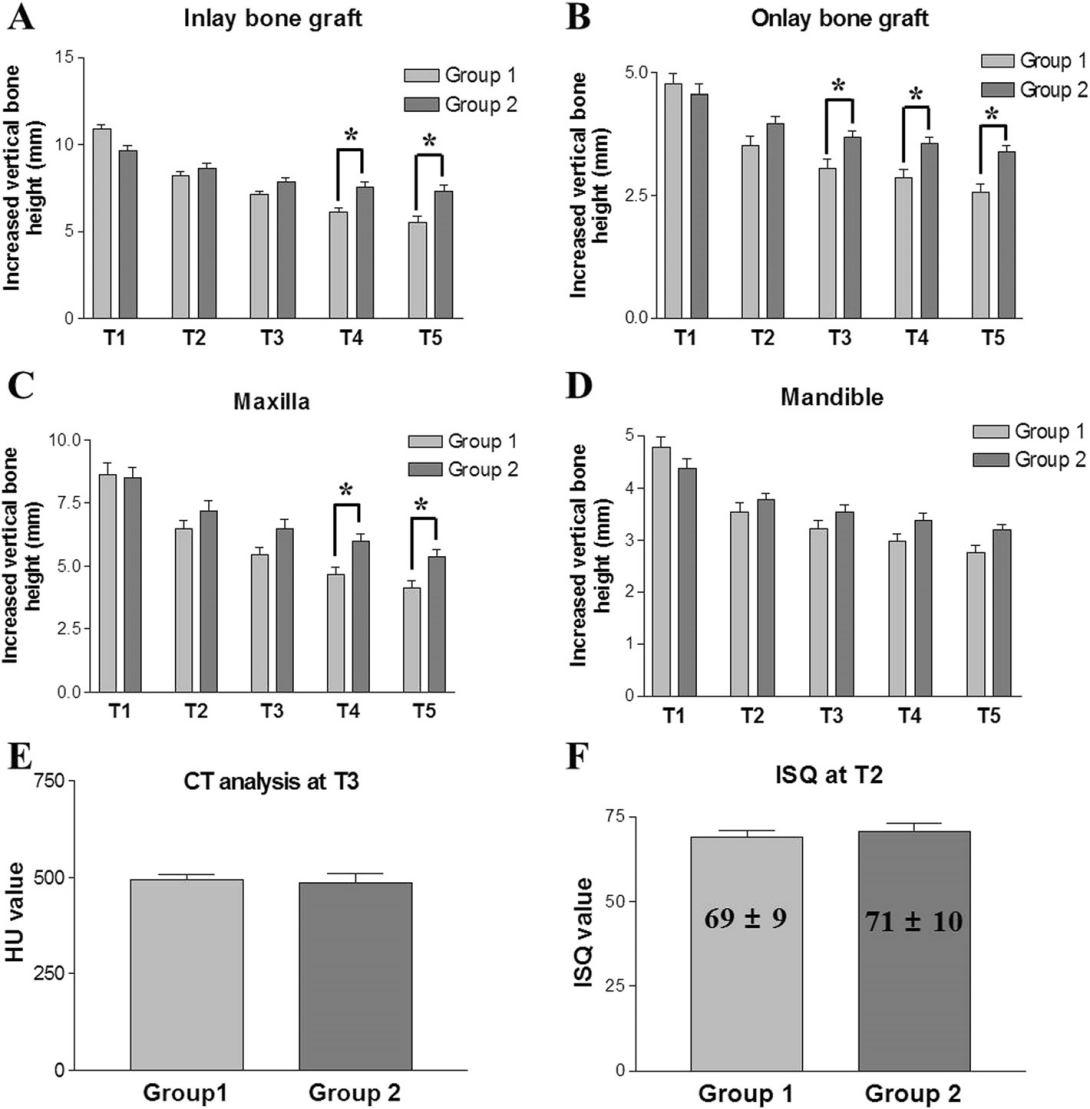
Comparison of the changes in increased bone height after bone graft (**a-d**), analysis of radiological intensity (HU values) in the newly generated bone using CT views at T3 (**e**), and implant stability quotients (ISQ values) by resonance frequency analysis results at T2 (**f**). **a & b** In both inlay and onlay type bone grafts, Group 1 shows a more prompt vertical bone loss than Group 2; there is statistical difference in remaining bone height between the two groups at T4 and T5. The intraoral jaw bone graft group has more residual grafted bone height than the iliac bone graft group after 2–3 years postoperatively (*p* < 0.05). **c & d** Changes in the vertical bone height were compared in the maxillary and mandibular ridges. In the maxillary fixtures, the intraoral jaw bone graft group showed a statistically lower vertical bone resorption rate at T4 and T5 than the iliac bone graft group (*p* < 0.05). A similar tendency for vertical bone resorption was observed in the mandibular fixtures, with no statistical difference between the groups (*p* > 0.05). **e** CT views at T3 (1 year postoperatively) reveal similar HU values in the newly generated bones between the two groups (*p* > 0.05). **f** Implant stability tests by resonance frequency analysis at T2 (5–6 months postoperatively) exhibit similar ISQ values between the two groups (*p* > 0.05). Data represent mean ± standard deviation, and an asterisk (*) indicates a significant difference between Groups 1 and 2 (*p* < 0.05)

Using panoramic views, the vertical alveolar bone height was measured in simultaneously placed implant sites at each time point (Fig. 3). In subantral inlay bone graft sites, the mean increased vertical bone height immediately after the operation (T1), calculated in comparison with preoperative alveolar bone height (T0), was 10.8 ± 0.9 mm in Group 1 and 9.6 ± 1.0 mm in Group 2. Following this, in Group 1, the augmented bone height promptly decreased to 8.2 ± 0.9 mm 6 months postoperatively (T2), and continuously reduced to 5.6 ± 0.9 mm by T5. However, in Group 2, there was no statistical difference in the mean increased bone height between T1 (9.6 ± 1.0 mm) and T2 (8.7 ± 1.0 mm); there was a gradual reduction to T5 (7.3 ± 1.2 mm). Therefore, in subantral inlay bone grafts, the ratio of residual grafted bone height at T5, compared with the increased bone height at T1, was 51.9 % in Group 1 and 76.0 % in Group 2 (*p* < 0.05) (Fig. 5a & Table 2). Similarly, in onlay-type bone grafts, the mean vertical alveolar bone increase at T1 was 4.9 ± 0.9 mm in Group 1 and 4.5 ± 0.7 mm in Group 2; at T5, this decreased to 2.6 ± 0.7 mm in Group 1 and 3.4 ± 0.5 mm in Group 2. The ratio of residual grafted bone height at T5 was 53.1 % in Group 1 and 75.6 % in Group 2 (*p* < 0.05) (Fig. 5b & Table 2). Changes in increased vertical bone height were compared between the two bone graft groups. In both inlay and onlay type bone grafts, Group 1 showed more rapidly vertical bone loss than Group 2; there was a statistical difference in remaining bone height from T3 and T4 between the two groups (*p* < 0.05) (Fig. 6a & b). In addition, the changes in the vertical bone height of Groups 1 and 2 were compared in the maxillary and mandibular ridges. In the maxillary fixtures, the intraoral jaw bone graft group showed a significantly lower vertical bone resorption rate at T4 and T5 than the iliac bone graft group (*p* < 0.05). The vertical bone resorption tendency was similar for the mandibular fixtures, with no statistical difference between the groups (*p* > 0.05) (Fig. 6c & d). These results indicate that jaw bone grafting showed a slower vertical bone resorption tendency of the grafted bone than the iliac bone grafting, in both subantral inlay and onlay bone grafts, resulting in greater residual bone height during long-term follow-up periods. In addition, CT scans were taken at T3 (1 year postoperatively) in 25 patients (15 in Group 1 and 10 in Group 2), and comparison of measured radiological intensities (HU values) in the newly generated bone showed no statistical difference between the two groups (Fig. 4 and 6e).

### Analysis of ISQ value and peri-implantitis incidence

**Fig. 7:**
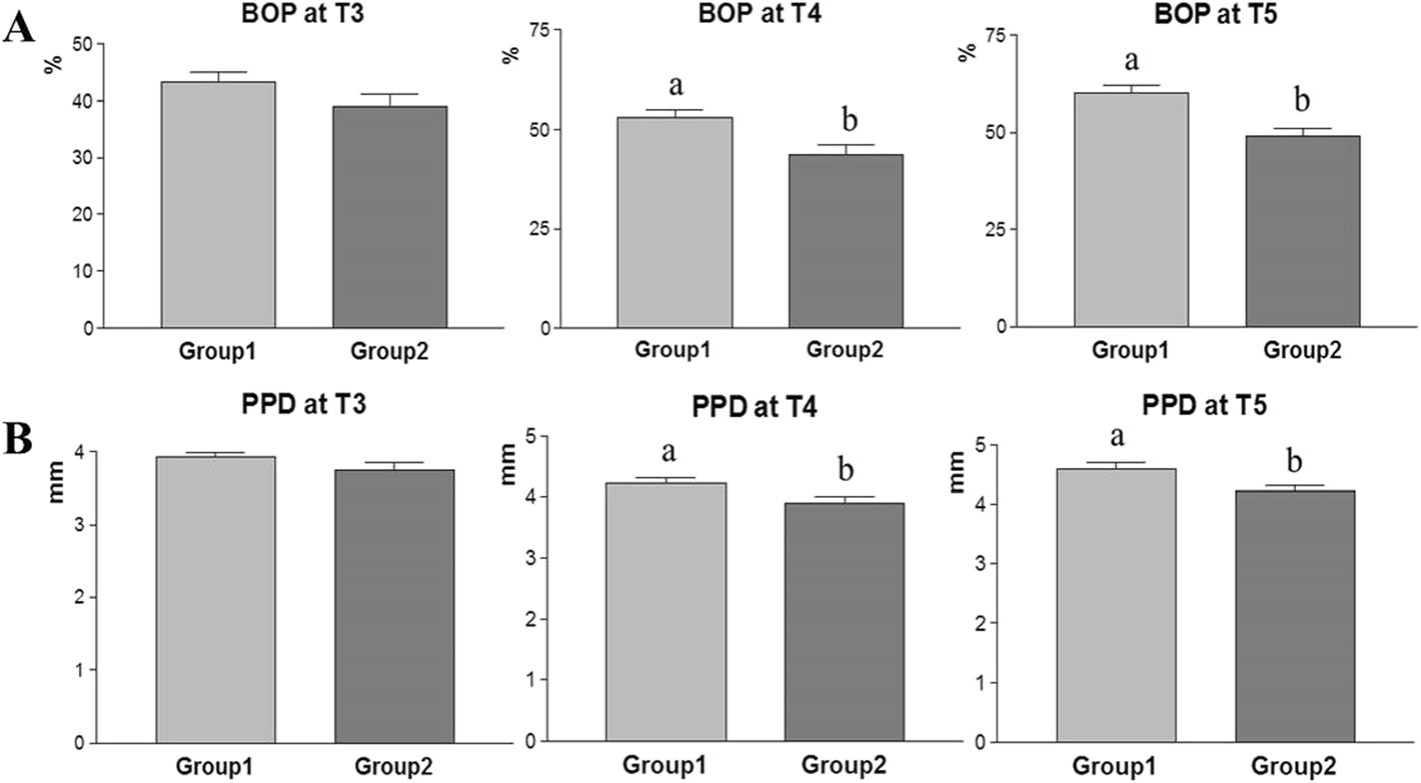
Graphs present the percentage BOP (**a**) and PPD (**b**) in the two groups during the follow-up period. Within 2 years postoperatively (T3), there is no significant difference in BOP and PPD between the two groups. However, the intraoral jaw bone graft group (Group 2) has a significantly lower percentage of BOP and PPD than the iliac bone graft group (Group 1) at T4 (2–3 years postoperatively) and T5 (>3 years postoperatively). Different letters denote statistical differences between groups (*p* < 0.05)

The mean ISQ value was 69 ± 9 in Group 1 and 71 ± 10 in Group 2; there was no statistical difference between the two groups (Fig. 6f). During the follow-up period (T3–T5), the percentage of BOP and PPD was measured for each implant site. Within 2 years post-operatively (T3), there was no significant difference in BOP and PPD between the two groups. However, the jaw bone graft group (Group 2) had a significantly lower percentage of BOP and PPD than the iliac bone graft group (Group 1) at T4 (2–3 years postoperatively) and T5 (more than 3 years postoperatively) (*p* < 0.05). These results indicate that intraoral jaw bone grafts could provide stronger resistance against peri-implantitis than iliac bone grafts (Fig. 7).

## Discussion

In the literature, delayed dental implantation is generally recommended after alveolar ridge augmentation in atrophic ridges [12–15]. Implant placement on consolidated bone may increase implant stability and lead to better prosthetic outcomes [13]. However, other studies have reported favorable results regarding implant success rates and esthetic prostheses after simultaneous implant placement with bone grafting in severely atrophic alveolar ridges in patients with a residual height of less than 4 mm [5–8]. This single-stage procedure reduces the number of surgical interventions and the total treatment time for patients [13]. Some researchers have reported that, if there is no mechanical stimulation on the grafted bone for 6 months after grafting, the grafted bone starts to be resorbed and its volume is reduced [16]. This could be explained by the mechanostatic theory that emphasizes mechanical stress for bone generation [17, 18]. The mechanical strain drives bone cells to change the bone structure. The magnitude of loading, the type and rate of physical activity, and the number of repetitions are pivotal mediators of physical activity on bone [17]. Similar concepts are applicable to jawbones since appropriate occlusal forces involved in remodeling basal bones are transmitted to the bone through teeth and periodontal ligaments [19]. Therefore, implant placement at the optimal time and application of appropriate occlusal force are important to promote the corticalization and maturation of newly formed bone [18, 20, 21]. This supports the theory that simultaneous dental implant placement and bone grafting could reduce early postoperative grafted bone resorption rate. In the present study, we postulated that the application of proper occlusal forces beginning at 6 months after surgery (implantation and bone graft) would reduce fatty changes in the grafted bones and promote consolidation of the new bones. Indeed, fatty changes in the newly generated trabecular bones are usually observed if occlusal force is not applied at the optimal time after bone grafting in the maxilla and mandible [18, 22].

In bone grafting techniques, autologous bone graft is considered the gold standard for reconstruction of bone defects and offers various advantages compared with xenogenic, allogenic, or synthetic bone grafting such as faster bone consolidation, higher regenerated bone quality, and reduced immune and inflammatory reactions [23, 24]. Since autologous bone grafts can transplant healthy osteoblasts and osteogenic proteins as well as bone matrix, ridge augmentation with autologous bone grafts has been strongly recommended in cases of severely atrophic alveolar ridges for safe placement of dental implants [16, 25, 26]. However, pure autologous bone grafts, especially particulate bone, have shown a greater volume reduction after consolidation of grafted bone, even though they have a higher viability [15]. A mixture of autologous bone and allogenic or xenogenic bone can be used as substitute graft materials to overcome the limitations of the autologous-only or allogenic-only graft method. In the literature, the mixed bone graft shows favorable results by increasing alveolar ridges and sinus floors [27–29]. These grafts may exhibit a synergistic activity to stimulate osteogenesis; autologous bone can provide sound osteoblasts and various osteogenic proteins or cytokines, while allogenic or xenogenic bone offers an abundant bone matrix that maintains the space during new bone generation [27–29]. In the present study, a 2:1 ratio of autologous bone and DBM was used for ridge enhancement; this appeared to, not only increase the graft material volume, but also reduced the grafted bone shrinkage volume and increased osteogenic activity.

The donor site of autologous bone is also a major consideration for successful autologous bone grafting, and affects the long-term resorption rates of grafted bones and implant success rates. In situations requiring larger bone volumes, the iliac crest is usually selected as the donor site for autologous bone. This has some advantages for reconstructing jawbones including a greater thickness, for reconstructing large intraoral bone defects, and its extraoral bone harvesting that can be set up as a two-team approach to reduce surgery time [13]. However, the most serious problem associated with the iliac free bone graft is a higher bone resorption rate during the early healing phase [8, 23]. Many researchers have reported higher bone volume changes in iliac bone graft sites than in calvarial or intraoral jaw bone (chin or ramus bone) graft sites [11, 13, 30, 31]. In the literature, the long-term bone resorption rate for iliac bone graft is reported at 12 to 60 %, while the resorption rate of calvarial bone graft is 0 to 15 % [23, 31, 32]. Similarly, autologous bone harvested from the chin has shown greater mineralization and a lower resorption rate after transplantation in the alveolar ridges than those of autologous bone from the anterior or the posterior iliac crest [33]. The origin of the intraoral jaw bone was the same as the recipient sites. Furthermore, the calvarial bone and jaw bone are formed by membranous bone formation, while the ilium is generated by endochondral bone formation. These differences in bone formation mechanisms could influence bone resorption rates after grafting into jawbones, that are formed by membranous bone formation [30, 34].

In the present study, the grafted autologous iliac bone was compared with the intraoral jaw bone for evaluation of long-term stability of simultaneously placed implants and resorption rates of grafted bones. The implant stability (ISQ values) at T2 and the bone density (Hu values in CT view) at T3 revealed no differences between the two autologous bone graft groups. However, the jaw bone graft group exhibited slower vertical bone resorption rates and smaller percentages of PPD and BOP over long-term follow-up than the iliac bone graft group. The grafted iliac bone showed more prompt vertical loss than jaw bone; in particular, the largest vertical bone reduction was observed within 6 months after bone graft. These findings are comparable with the results of previous studies that indicated that jaw bone may be more suitable than iliac crest bone to augment alveolar bone volumes and to provide greater implant survival rates in atrophic ridge [34, 35]. The intraoral jaw bone appears to adapt and remodel with greater ease in recipient beds, and may provide a stronger resistance to peri-implantitis than the iliac bone. Further, the intraoral jaw bone was easily harvested by the intraoral approach from the area surrounding the surgical field of implant placement and therefore negated the need for mandatory general anesthesia [36]. In addition, the harvested bones from the chin, the mandibular ramus, and/or the maxillary tuberosity provided sufficient bone volume for 2:1 or 1:1 mixed bone with DBM; this can be grafted in the alveolar ridge as onlay- and/or inlay-types to cover the exposed fixtures and fill the sinus floor.

## Conclusion

In resonance frequency analysis, simultaneous implant placement and bone grafting with mixed bone grafts of autologous bone and DBM (v/v ratio: 2:1) provided sufficient initial implant stability to support dental prostheses 5 to 6 months after surgery. Two types of autologous bones that varied according to their donor sites (i.e., the iliac crest or the jaw bone) showed favorable clinical results, with similar long-term implant stability and overall implant survival rates. However, the grafted iliac bone showed more prompt vertical loss than jaw bone; the largest vertical bone reduction was observed within 6 months after bone graft. The jaw bone graft group had slower vertical bone resorption rates and lower peri-implantitis incidence during long-term follow-up than the iliac bone graft group. Furthermore, the jaw bone could be easily harvested from intraoral sites during implant surgery, without the need for an extra-surgical field, providing sufficient volume for the mixed bone with the DBM. The results of this study demonstrate that simultaneous dental implantation with autologous intraoral jaw bone grafting method may be reliable for the reconstruction of edentulous atrophic alveolar ridges.
